# Reinforced Fill Structure with Alternative Fill Materials: An Application of Geogrid Creep Strain Analysis Using Numerical Modeling

**DOI:** 10.3390/ma18061346

**Published:** 2025-03-18

**Authors:** Ahsan Rehman Khan, Gemmina Di Emidio

**Affiliations:** UGent Geotechnical Institute, Department of Civil Engineering, Ghent University, 9052 Gent, Belgium; gemmina.diemidio@ugent.be

**Keywords:** reinforced fill structure, alternative fill, geogrids, finite element analysis, strain, viscoelastic, creep

## Abstract

For many years, granular fill has been the preferred fill material in reinforced fill structures (RFSs) due to its high strength and drainage properties. However, the global scarcity of granular fill has necessitated the exploration of alternative fill materials. This study aims to evaluate the performance of three different alternative fill materials: (i) weak onsite fill (fill 1), (ii) lime-stabilized onsite fill (fill 2), and (iii) recycled construction and demolition (C & D) waste (fill 3). A finite element analysis (FEA) was conducted to assess the stability and horizontal displacement of an RFS and the long-term creep deformation of geogrid using viscoelastic (time-dependent) model in Plaxis. This RFS comprised a combination of wire mesh and geogrids, serving as primary and secondary reinforcement materials, respectively. The results indicate that fill 1, with low shear strength and stiffness, induces excessive lateral displacement and was unstable, making it unsuitable for RFS applications. In contrast, Fill 2 and Fill 3 achieve Eurocode-based safety factors of 1.12 and 1.19, respectively, while significantly reducing horizontal displacement. The long-term creep deformation analysis of geogrid in the case of fill 1 exceeds the prescribed serviceability strain limit threshold, while in the cases of fill 2 and fill 3, it conforms to the serviceability strain limit, which indicates effective mobilization of tensile resistance without excessive elongation. Finally, an analysis was conducted to optimize the geogrid length and to see its impact on cost and performance. The results revealed up to a 29% cost reduction while ensuring performance criteria. These findings validate lime-stabilized onsite fill and recycled C&D waste as viable, cost-effective alternatives to conventional granular backfill, ensuring not only stability and serviceability but also the long-term performance of geogrids in RFSs.

## 1. Introduction

Resource efficiency is essential in sustainable development. Due to increasing urbanization, a significant number of engineering projects, including buildings, bridges, and roads, are being built in mountainous areas and beside rivers [1,2,3,4]. Geosynthetics has been widely used in these type of projects for soil reinforcement due to their numerous benefits, such as improving strength and stiffness [5,6]. Some of the most widely used forms of geosynthetics are geotextiles, geogrids, geo-composites, geocells, geonets, and geofoams [7,8]. They serve one or more of the following purposes: filtration, separation, sealing lateral drainage, and reinforcement [9]. They are increasingly acknowledged as essential for sustainable infrastructure development due to their ability to decrease the carbon footprint associated with infrastructure projects by reducing the reliance on natural building materials [10]. The addition of geosynthetics as reinforcement in the retaining structures is due to withstanding the forces which tend to pull the backfill mass outward. By inserting the reinforcing elements in the soil, the stiffness of the soil can be enhanced, which causes a decrease in the deformation of the soil [11].

RFSs can be constructed using a combination of wire mesh and geogrids, which are commonly used as primary and secondary reinforcement materials [12]. Geogrids are utilized as primary reinforcements due to their ability to prevent the structure from experiencing any potential rupture surfaces, where wire mesh acts as a secondary reinforcement to provide stability at the facing [13]. Gabion walls, due to their weight, show dominating strength against hydraulic and active soil pressure. The gabions with wire mesh boxes are built by welding or twisting, and the boxes are filled with an inorganic material [14]. These are also more cost-effective when compared to traditional walls [15]. In the past few years, wire mesh has been widely used as a reinforcement material for stabilizing earth structures. These materials are usually utilized in combination with units that can provide a stone-facing surface [12]. Besides stabilization, they can also be utilized for the construction of riverbanks, erosion control measures, and landslide mitigation. They can be used to increase the wall height [16,17].

Geogrids are polymeric materials that tend to exhibit time-dependent properties when embedded in soil and exposed to tensile loads [18]. The viscoelastic characteristics of geogrids are susceptible to influencing the behavior of reinforced systems over the design lifetime, and hence long-term mechanical properties of geosynthetic as well as soil geosynthetics interactions are of critical importance [19].

Another major advantage of using geosynthetics is their ability to allow the use of low-quality materials as a fill in an RFS, especially in those areas where there is a scarcity of granular material [20]. Various studies have highlighted the importance of factors such as the type of fill material, its properties, and compaction in determining the performance of RFSs [21]. Different case studies have shown that poor or inconsistent compaction can lead to the failure of RFS structures [22,23].

The quality of the backfill, whether cohesive or cohesionless, plays a crucial role in the stability and displacement characteristics of an RFS [24]. Walls with cohesive backfill have been observed to exhibit larger displacement compared to those with cohesionless backfill [24]. Moreover, the properties of the geogrid, including stiffness, length, and spacing, are also influential factors in the behavior of RFSs [25]. The reinforcement stiffness, backfill type, facing type, and peak ground acceleration have been identified as critical parameters affecting its cyclic response [26]. Furthermore, the elastic moduli of the fill have been shown to significantly impact the performance [27].

The selection of building materials impacts key factors during production, including resources used, embodied energy, energy content, greenhouse gas emissions, carbon storage, and material costs [28]. With growing awareness of the emissions associated with building processes, there is a shift towards designing conservation measures that ensure high performance in terms of both health and safety [29,30]. As the scarcity of conventional materials continues to be a concern, there is an increasing need to replace them with alternatives to address these challenges [31]. In this context, the suitability of recycled C&D material as an alternate fill in the construction of RFSs has raised the attention of researchers [32]. Backfill costs typically range from 50% to 75% of the entire retaining structure cost [33]. The Federal Highway Administration recommends that backfill material with less than 15% fine content is the most suitable for soil [34]. According to the National Concrete Masonry Association [35] guidelines, soil particles of up to 35% are permitted, as long as proper drainage concerns are taken into account. Due to the scarcity of high-quality backfill material near construction sites, it would be financially impractical to use granular backfill even if the conveyance is available.

In spite of these concerns, marginal fill has the potential to enhance matric suction and consequently increase shear strength and hence the effectiveness of RFS walls increases [36]. However, due to global climatic changes, an unanticipated quantity of rainfall may cause an increase in infiltration, which leads to a decrease in suction. Several studies [22,37,38] underscore the importance of considering the decrease in interface shear strength caused by suction loss due to rain-induced wetting when employing marginal fill, particularly in areas prone to intense monsoon rainfall. Koerner and Koerner in 2011 [33] mentioned that out of the 82 cases in their database, 68% failure was identified due to improper drainage.

The use of locally sourced materials as a fill in reinforced fill structures (RFSs) offers significant economic and sustainable advantages [39]. However, challenges and uncertainties persist regarding their optimal use and configuration. Key factors such as deformation behavior and the long-term performance of geogrids must be thoroughly evaluated to ensure the structural integrity and stability of the RFS. This study will investigate the impact of three different fill materials, which include onsite fill material as fill 1, onsite fill material stabilized with lime as fill 2 and recycled C&D material as fill 3, on the stability of the RFS, horizontal displacement, and short-term and long-term performance of geogrids in terms of strain. A unique aspect of this research is its use of a viscoelastic time-dependent model in Plaxis V24 to assess the long-term creep strain behavior of geogrids, which has not been explored in previous studies. This practice not only helps with utilizing onsite materials that may otherwise go to waste but also offers economic and sustainability advantages. By incorporating these local materials into the construction process, the need for new materials is reduced, leading to cost savings and minimizing the environmental impact associated with sourcing and transporting materials over long distances. Furthermore, utilizing onsite materials for filling can contribute to a significant reduction in the project’s carbon footprint. By avoiding the transportation of waste materials and reducing the reliance on new resources, construction projects can achieve substantial environmental benefits. This aligns with the principles of sustainable construction practices, where the emphasis is on minimizing waste generation, resource consumption, and energy usage throughout the project lifecycle.

## 2. Materials and Methods

**Figure 1:**
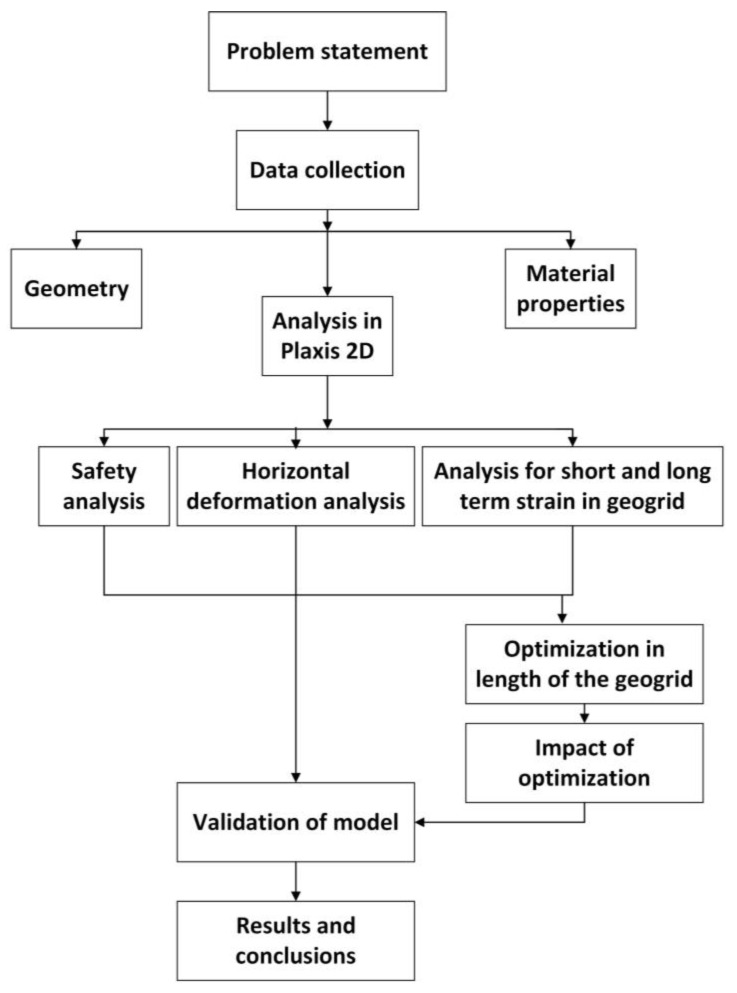
Research methodology.

The complete research methodology is illustrated in Figure 1 as shown below.

### 2.1. Numerical Modeling of Reinforced Fill Structure

**Figure 2:**
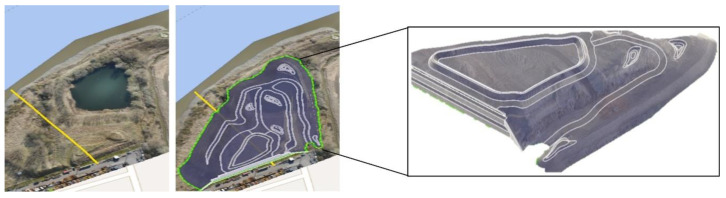
Hydropower hill having 12 m high reinforced fill structure.

**Figure 3:**
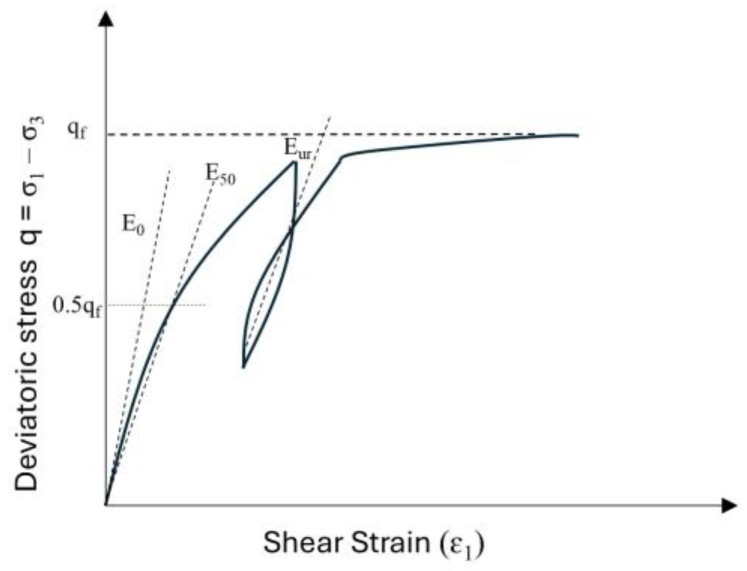
Representation of the hardening soil model.

This study discusses the stability of the hydropower hill having weak underground strata, as shown in Figure 2. A 12 m high RFS structure is employed for this purpose. Plaxis-2D is used to design and analyze this structure. In this study, we aimed to investigate the stability of the RFS, horizontal displacement, and the short-term and long-term performance of geogrids in terms of strain using FEA. The geometric configuration of the RFS in our investigation is shown in Figure 3. The foundation strata supporting the wall are notably weak (due to confidentiality agreements, some details of the soil strata have been omitted from this publication). The RFS comprises a combination of wire mesh and geogrids, serving as primary and secondary reinforcement materials, respectively. Geogrids are employed as primary reinforcements as they are instrumental in preventing potential rupture surfaces within the structure. Additionally, wire mesh acted as secondary reinforcement, providing strength at the facing [13]. 

The geogrid reinforcements possess a maximum tensile strength of 300 kN/m being employed. A vertical spacing of 1 m between the reinforcements is selected, and the axial stiffness of the geogrids is calculated. The construction process of reinforced walls is simulated using staged construction methods. Investigation under operational stress conditions involved analyzing the distribution of strain and identifying locations of maximum tensile load in the reinforcements and examining post-construction deformation. Usually, geogrids are susceptible to experiencing greater displacements over time as a result of creep deformations under sustained loads. The primary cause of stiffness loss in an RFS is attributed to creep behavior. In this study we will also find the long-term creep deformation of the geogrid using the viscoelastic (time-dependent) model. For this purpose, we need to calculate the short-term stiffness (EA_short_) and the long-term stiffness (EA_long_) of the geogrid, maximum force N_p_, and the retardation time t_r_.

### 2.2. Material Properties for the Finite Element Method

Table 1 presents the soil properties, which include foundation strata, and three different types of fill materials, i.e., onsite material as fill 1 and onsite material stabilized with lime as fill 2, as demonstrated in our previous study [40], while recycled C&D material as fill 3 is taken from [41]. Table 2, Table 3 and Table 4 present the properties of geogrid, gabions, and wire mesh, respectively. The strata exhibited complete heterogeneity and a mixed geological origin. Specific details of the soil strata have been omitted from this publication due to confidentiality agreements with our industry partner. This decision was made to respect the proprietary nature of the information while ensuring that the integrity and scientific rigor of the research remain intact.

The hardening soil model is used to replicate the behavior of soil as it takes into account the stress dependency of soil stiffness and a better estimate of deformation analysis. The actual stiffness of soil is non-linear and the hardening soil model is able to predict this behavior. The model considers the non-linear stress–strain characteristics of soil, including phenomena such as soil softening and soil hardening. The stress–strain relationship in a triaxial compression test is represented by the hyperbolic function [42], as shown in Figure 3. The model shows a decreasing stiffness and irreversible plastic strains simultaneously when subjected to deviatoric loading. It incorporates three distinct input stiffness values for soil: the triaxial stiffness E_50_, the triaxial unloading stiffness E_ur_, and the edometer loading stiffness E_oed_. The hardening soil model differs from the Mohr–Coulomb model by adding the stress-dependent nature of stiffness moduli.

**Figure 4:**
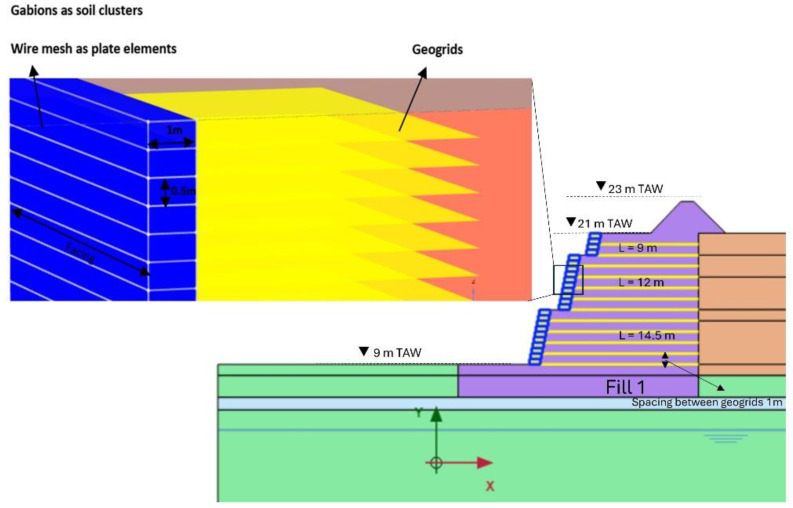
Numerical model of a reinforced fill structure in Plaxis.

As shown in Figure 4, the facing of the wall is composed of gabion encased in wire mesh, so gabion block is modeled as a soil cluster. Here the geogrids serve as the primary reinforcement, while wire mesh acts as a secondary reinforcement [12].

### 2.3. Modeling of Geogrids

**Figure 5:**
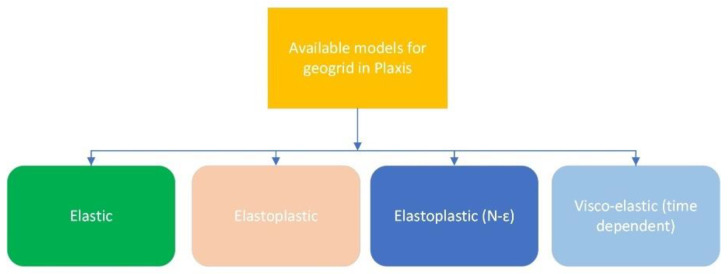
Types of models for geogrid in Plaxis.

A geogrid element is a type of line structure that extends in the out-of-plane direction and has no flexural stiffness [43]. A geogrid element has two translational degrees of freedom per node (ux, uv) and can only undergo tension, not compression. In Plaxis, the geogrid can be modeled as shown in Figure 5:

For the elastic model, we can specify the normal elastic stiffness of a geogrid element:

- EA_1_: normal elastic stiffness (in plane);
- EA_2_: normal elastic stiffness (out of plane).

If the geogrid material is set to the elastoplastic option, the following strength parameters would also be required:

- N_p,1_: maximum force (in plane);
- N_p,2_: maximum force (out of plane).

If the geogrid material is set to the elastoplastic (N − ε) option, a strain-dependent strength is specified through a table, as follows:

- N_1_ − ε_1_: the strain-dependent strength diagram (in plane);
- N_2_ − ε_2_: the strain-dependent strength diagram (out of plane).

If time-dependent geogrid interactions are to be considered, i.e., the strength-reduction effect of time (creep), then the viscoelastic (time-dependent) model should be chosen. Here, the following stiffness values should be specified:

- EA_1_, short: normal elastic stiffness during an instantaneous (initial) strain increment (in plane);
- EA_2_, short: normal elastic stiffness during an instantaneous (initial) strain increment (out of plane);
- EA_1_, long: normal elastic stiffness during a long-term (infinite) strain increment (in plane);
- EA_2_, long: normal elastic stiffness during a long-term (infinite) strain increment (out of plane);
- N_p,1_: maximum force in one direction (in plane);
- N_p,2_: maximum force in two directions (out of plane);
- tr: retardation time.

### 2.4. Modeling of Geogrid for Long-Term Creep Deformation

**Figure 6:**
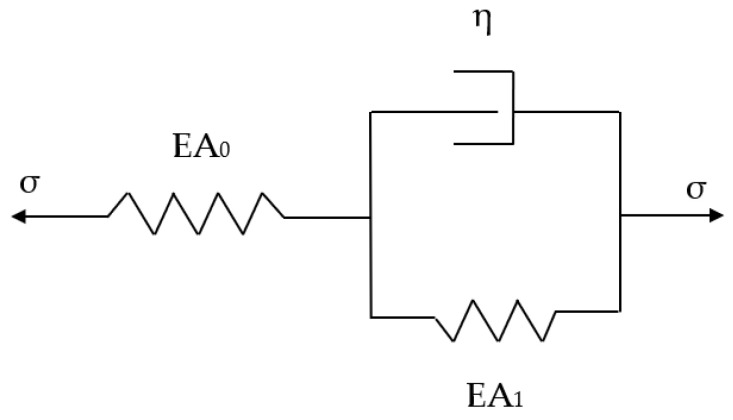
Kelvin–Voigt single element representation.

The design life of the reinforcement can vary from a few months to up to 120 years depending upon the type of structure [44]. The principal function of reinforcement is to withstand tensile loads. Geogrids are polymeric materials that tend to exhibit time-dependent properties when embedded in soil and exposed to tensile loads [45]. Polymers are nonlinear viscoelastic materials and exhibit time-dependent properties, particularly under constant load, where they experience creep, i.e., an increase in strain over time. As time progresses, the tensile strength of the polymer reinforcement decreases. This causes the material’s tensile strength to decrease from an initial short-term value to a lower creep rupture strength by the end of the intended design life. Plaxis uses the Kelvin–Voigt model to determine the long-term creep deformation of the geogrid [43]. This model combines elastic and viscous elements to obtain a physical interpretation of stress–strain relationships, as shown in Figure 6. Equation (1) as per [46] is be used to determine the design strength of the geogrids:

(1) $$T_{\mathrm{all}}=\frac{T\mathrm{ult}}{{\mathrm{RF}}_{\mathrm{CR}}\times {\mathrm{RF}}_{\mathrm{ID}}\times {\mathrm{RF}}_{W}\times {\mathrm{RF}}_{\mathrm{CH}}\times f_{s}}$$

where T_ult_ = short term ultimate tensile strength;

RF_CR_ = reduction factor due to creep;
RF_ID_ = reduction factor for installation damage;
RF_W_ = reduction factor due to weathering;
RF_CH_ = reduction factor to allow for reductions in strength due to chemical and biological effects at the design temperature;
fs = factor of safety for accounting the statistical variation in the reduction factors calculated.

The reduction factor for creep is derived from the time-creep degradation curve or the isochronous curve of the relevant material. When strain is not a limiting factor, the remaining strength after a given number of years can be assessed using the time-creep degradation curves. However, if strain is a limiting factor, the isochronous curves are used to calculate the remaining strength based on the limiting strain and the structure’s design life.

The creep strain ϵ(t) for the Kelvin–Voigt model under a constant stress σ0 is given by Equation (2) [47]:

(2) $$\epsilon (t)=\frac{\sigma_{0}}{E}(1-e^{\frac{-t}{\tau }})$$

where

σ_0_ = applied stress (constant);
E = elastic modulus of the spring;
τ = retardation time;
t = time.

Considering the Kelvin–Voigt element as shown in Figure 5,

(3) $$t_{\mathrm{retardation}}=\frac{\eta_{1}}{E_{1}}$$

EA_0_ = EA_short_ (4)

(5) $$\frac{1}{EA_{0}}+\frac{1}{EA_{1}}=\frac{1}{EA_{long}}$$

(6) $${\mathrm{EA}}_{1}=\frac{1}{\left(\frac{1}{EA_{long}}-\frac{1}{EA_{0}}\right)}$$

(7) $${\mathrm{EA}}_{\mathrm{short}}=\frac{1}{EA_{0}}$$

(8) $${\mathrm{EA}}_{\mathrm{long}}=\frac{F}{\epsilon_{\mathrm{long}}}$$

(9) $$\epsilon_{\mathrm{short}}=\frac{F}{{EA}_{short}}$$

(10) $$\epsilon_{\mathrm{long}}=\frac{F}{{EA}_{long}}$$

where η is viscous damping, and EA_0_ and EA_1_ are internal stiffness used in the geogrid.

**Figure 7:**
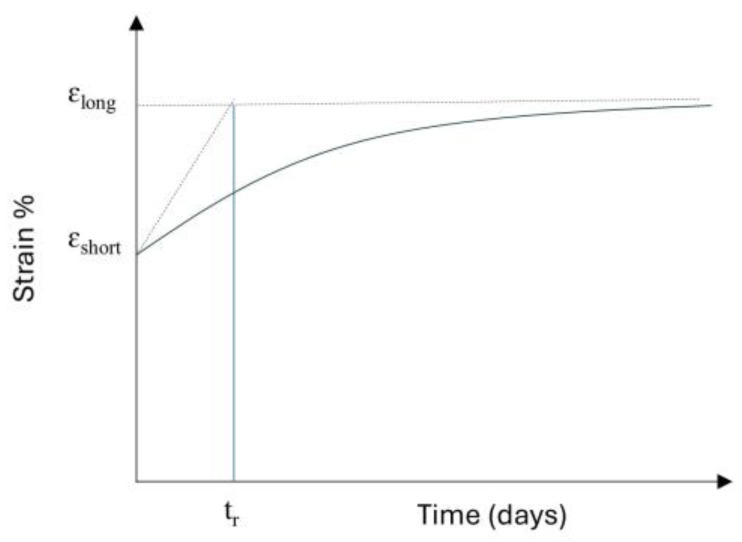
Evaluation of a retardation time in a strain vs. time curve in a creep test.

Isochronous curves illustrate the relationship between tensile load and strain at various time intervals. These curves help designers determine both the initial and post-construction strains for polymer reinforcements under specific stress levels, ensuring that strain remains within the prescribed design limits. According to BS 8006 [48], the post-construction strain limits vary depending on the type of structure. For bridge abutments, the maximum allowable strain is 0.5%, while reinforced fill structures can tolerate up to 1%. In cases where deformation is less critical, such as for slopes, strains up to 5% may be acceptable. Thus, the retardation time can be evaluated as shown in Figure 7. The retardation time is then defined as the time required for the strain, under a state of constant stress, to reach its maximum value if the strain rate remains constant and equal to the initial value. In other words, the retardation time is the necessary time to stop the creep process if the strain rate remains constant and equal to the initial value. The lower its value, the faster the creep process occurs, and the material is classified as less viscous. The retardation time provides the time estimate required for the creep process to approach its end. Most of the creep process occurs within the retardation time.

### 2.5. Interface Coefficient

Reinforcing elements decrease horizontal displacement and increase the stability of the RFS [49]. These reinforcements work by confining the soil mass, effectively distributing applied loads, and improving the overall performance of the structure. So, the interaction between the geogrid and soil plays a vital role in the stability of the structure. To replicate this interaction in modeling the reinforced fill structure, an interface is provided between the soil and the reinforcement. This interface facilitates the accurate transfer of these loads, ensuring that the behavior of the wall under different loading conditions is properly represented. Understanding shear resistance along the interface is important for predicting the sliding and deformation behavior of the RFS. Hence, these interaction parameters necessitate consideration in design calculations [50]. Usually, pull-out tests are used to determine the interface between the soil and the reinforcement in an RFS. The interaction between soil and geosynthetics is influenced by various factors such as the structural, geometrical, and mechanical characteristics of the geosynthetics, as well as the mechanical properties of the soil, along with considerations related to boundaries and loading conditions [51]. The performance of an RFS is significantly influenced by interactions between its different components, i.e., fill, geogrid, and facing elements [52,53,54]. Different methods are used to simulate these interactions [49,50]. Because these simulated interactions might alter the projected performance of RFSs, it is critical to use appropriate interaction-simulation techniques in a numerical study [55].

A specific interface coefficient value was adopted, following the manufacturer’s guidelines, and was modeled in Plaxis software. A table listing various types of fill materials and their corresponding interface coefficient values, as recommended by the manufacturer, is shown in Table 5. For confidentiality reasons, the specific interface values are not included in this publication.

## 3. Validation of Model

For the purpose of validation, the test results on a full-scale wall setup at Royal Military College, Cananda, reported by Bathurst and Walters [56,57], were used to calibrate our finite element model.

**Figure 8:**
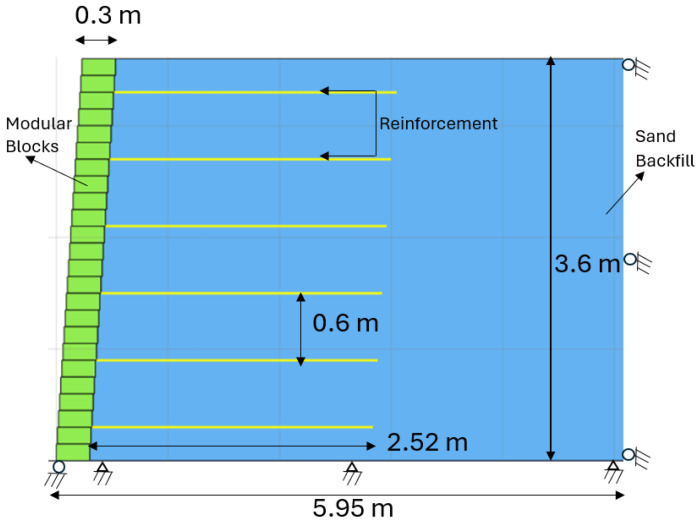
Full-scale wall modeled in Plaxis-2D.

**Figure 9:**
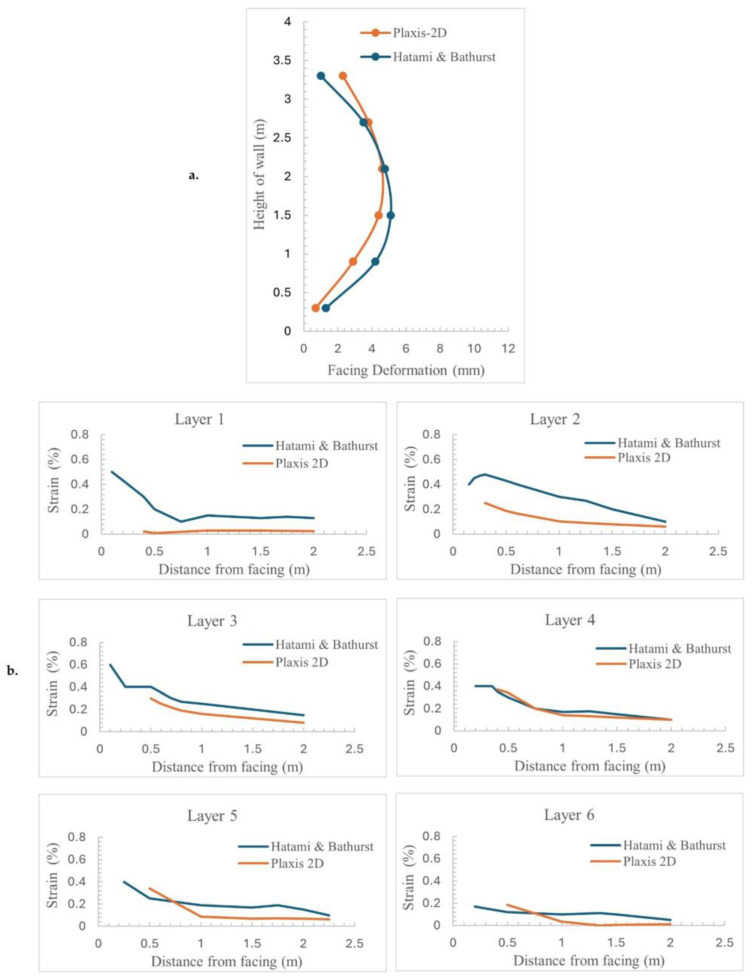
(**a**) Facing deformation of the wall along its height. (**b**) Strain (longitudinal) in %, on different layers of the wall along the length of the geogrid.

Using Plaxis-2D software, a model is constructed to represent a 3.6 m high wall with reinforcements measuring 2.52 m in length and spaced at 600 mm intervals, as shown in Figure 8. The properties of the soil, modular block, and reinforcements are mentioned in Table 6, Table 7 and Table 8, respectively, and adopted from documented sources [27,57]. The technique of staged building is used to replicate the step-by-step installation of the soil layer. Validation of the model involved analyzing the deformations and strains experienced by the reinforcements. The diagram showcases the deformation patterns of the reinforcement layers and the strain distribution along their axis. Figure 9 illustrates that the facing deformation and strain in the reinforcements, obtained through numerical simulations, correspond closely with findings from experimental studies.

## 4. Results and Discussion

A large number of simulations were conducted using FEA to assess the stability and horizontal displacement of an RFS as well as the long-term creep deformation of the geogrids using viscoelastic (time dependent) model in Plaxis.

### 4.1. Stability Analysis

**Figure 10:**
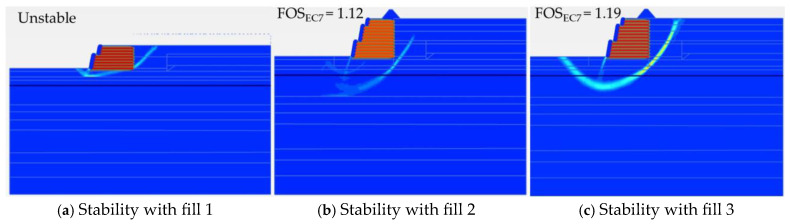
Stability analysis.

FEA carries out safety factor analysis by continuing to reduce the shear strength of the soil until failure is triggered. The safety factor is then obtained by dividing the original shear strength parameter by the last shear strength parameter that causes failure. In all these stability analyses, the safety factor has been determined using the design approach methodology prescribed by the Eurocode in Plaxis. Figure 10a–c present the results of the stability of the RFS using onsite fill material as fill 1, onsite fill material stabilized with lime as fill 2, and recycled C&D material as fill 3. Among the three tested fill materials, fill 1 exhibits the lowest cohesion, friction angle, and stiffness, resulting in lower shear strength and greater compressibility. This leads to higher lateral deformations, reducing the effectiveness of the geogrid reinforcement and compromising overall stability. In contrast, fill 2 and fill 3 demonstrate significantly higher stiffness and shear strength, limiting deformation and improving structural integrity. Fill 2, subjected to lime stabilization, exhibits more cohesion and friction angle, which enhances particle bonding and consequently improved shear strength. Fill 3, composed of recycled C&D aggregates, exhibits high shear strength and stiffness. Although its friction angle is slightly lower than that of fill 2, its performance remains comparable in terms of stability. Thus, the RFS is unstable with fill 1 and stable with fill 2 and fill 3, having a safety factor of 1.12 and 1.19, respectively, determined using the Eurocode partial-factor approach. 

### 4.2. Horizontal Displacement

**Figure 11:**
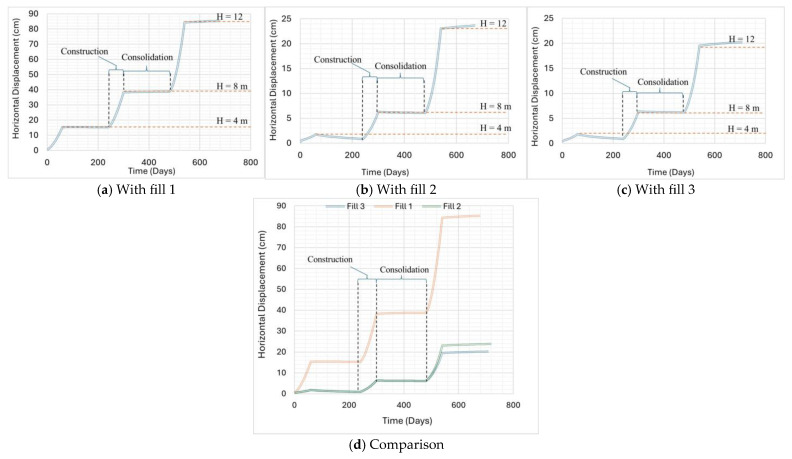
Change in horizontal displacement with time.

In this project, the allowable horizontal displacement is limited to 25 cm as per stakeholder recommendations. For each fill type, the staged construction process is carried out in phases, alternating between construction and consolidation. Each construction phase lasts for 60 days, followed by a consolidation phase of 180 days. The first stage involves constructing a 4 m high wall, followed by a consolidation phase. In the second stage, the wall height is increased to 8 m, and another consolidation phase is performed to account for the additional load. Finally, in the third stage, the wall is raised to 12 m, and a hydropower hill is added, as shown in Figure 11. This phased approach allows for a detailed analysis of the structure’s behavior under increasing loads and provides insight into the evolution of horizontal displacement over time. Among the different fill materials, fill 1 exhibits the highest horizontal displacement of 85 cm due to its low shear strength, low stiffness, and weak geogrid interaction, which reduces pullout resistance and prevents effective reinforcement engagement, resulting in greater lateral forces that further contribute to excessive movement at the toe. In contrast, fill 2 and fill 3 demonstrate significantly lower displacements of 23 cm and 20 cm, respectively, due to their higher shear strength and stiffness, which enable better distribution and resistance to deformation. Their stronger geogrid interaction enhances reinforcement efficiency by increasing interlock and reducing lateral displacement. These findings indicate that fill 1 is not suitable, while fill 2 and fill 3 provide stability by effectively minimizing deformation and enhancing overall structural performance.

### 4.3. Strain in the Geogrid

#### 4.3.1. Short-Term Strain

**Figure 12:**
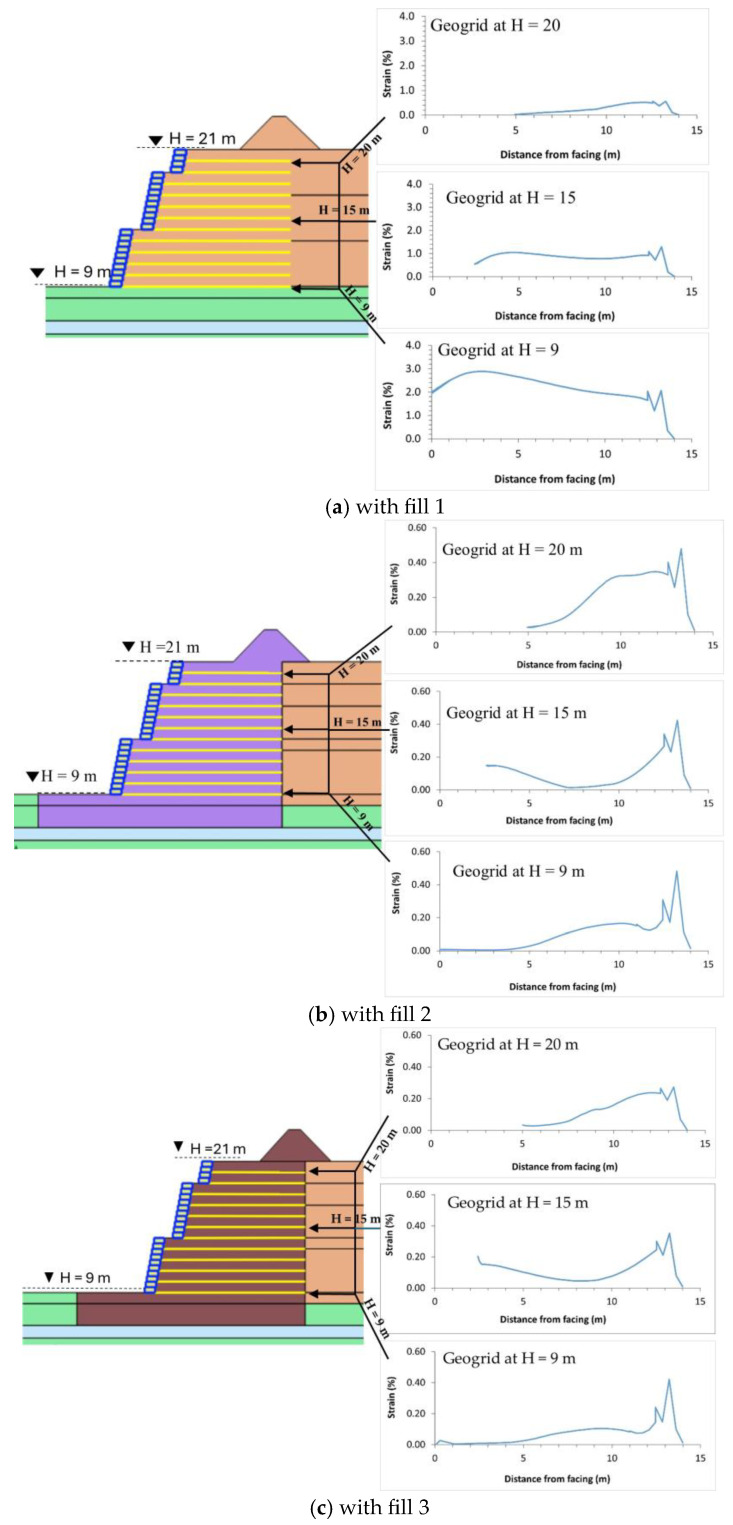
Strain envelope along the length of the geogrid.

Figure 12a–c illustrate the strain envelope along the entire length of the geogrid at three different heights of 9 m, 15 m, and 20 m for fill 1, fill 2, and fill 3, respectively. It is noted that a rise in vertical stress beneath the hydropower hill induces greater lateral pressure, consequently increasing the mobilized tensile force. This phenomenon is particularly pronounced beneath the hydropower hill. Consequently, there is an increase in the strain endured by the geogrid. Figure 12 also illustrates a trend where strain escalates along the geogrid’s length, peaking beneath the hydropower hill. These results are in good agreement with [58].

#### 4.3.2. Long-Term Strain

**Figure 13:**
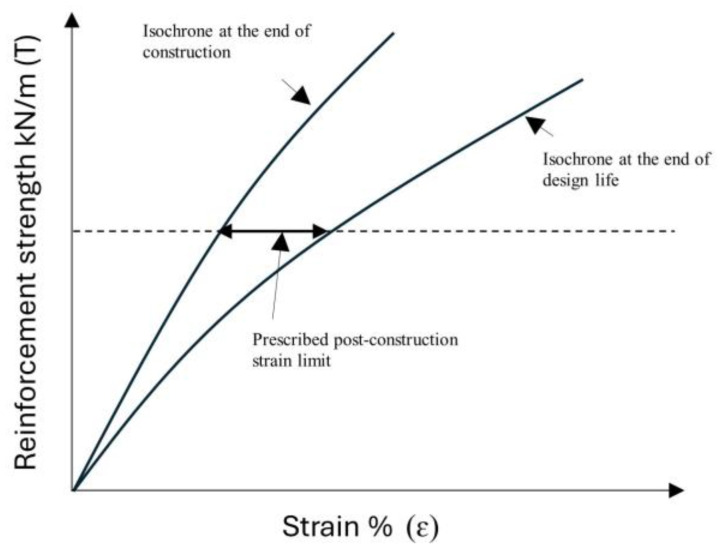
Prescribed post-construction strain limit assessment.

Polymeric reinforcement exhibits time-dependent behavior under tensile loads. The viscoelastic characteristics of geogrids are susceptible to the influence of the behavior of reinforced systems over the design lifetime. As these materials experience a decrease in short-term tensile stiffness due to creep, the long-term mechanical properties of the geosynthetic material and its interaction with the soil become critical. The strain occurring between the end of construction and the end of the selected design life should conform to the serviceability limit state, as shown in Figure 13. Hence, to adapt the serviceability limit state, the post-construction strain should not exceed the values given in Table 9. As per the British code for retaining walls, the prescribed post-construction strain limit, i.e., the difference between the long-term strain and short-term strain, should less than 1%.

**Figure 14:**
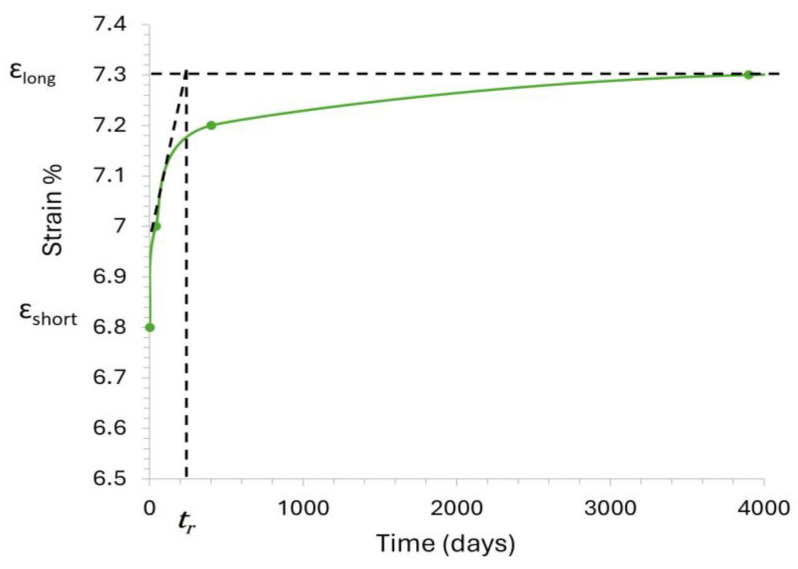
Strain versus time in a creep test of the used geogrid (from isochronous curve).

To find out the prescribed post-construction strain limit, we need to calculate the short-term axial stiffness (EA_short_) and long-term axial stiffness (EA_long_) of the geogrid. Using Equation (1), we can calculate the allowable tensile strength (T_all_). Once T_all_ is determined, the axial stiffness of the geogrid can be obtained by dividing its allowable tensile strength with its corresponding allowable strain. The retardation time can then be determined by analyzing the isochronous curves, which represent the relationship between tensile load and strain at various time intervals. These curves enable designers to assess both the initial and long-term strains of polymer reinforcements under specific stress conditions. By using the isochronous curves, a strain versus time curve for a given stress level can be generated. The initial creep behavior, observed at short time scales, is typically linear and can be approximated as a straight line. This linear segment is then extrapolated to estimate the strain progression if the initial rate of creep continued indefinitely. The retardation time corresponds to the point at which the extrapolated initial creep strain converges with the long-term creep strain, representing the duration required for the linear extrapolation to reach the steady-state creep level, as shown in Figure 14. From the isochronous curves, we calculated the retardation time, which approximately comes out to be 100 days. To better estimate the effect of retardation time, we consider two more times, i.e., 210 days and 365 days, to observe how different time intervals affect the analysis and results.

The prescribed strain limit of the geogrid exceeds 1% in the case of fill 1, as shown in Table 10, due to various factors which include poor fill–geogrid interaction; poor shear strength properties, i.e., low cohesion and a low friction angle; and lack of sufficient lateral resistance to prevent excessive geogrid deformation. This results in poor interlock and slippage between the soil and the geogrid, leading to higher strain. Additionally, fill 1′s high compressibility contributes to increased lateral displacement, which, in turn, raises the tensile demand on the geogrid. As the fill continues to deform over time, the geogrid undergoes sustained stretching, leading to elevated long-term strain. In contrast, fill 2 and fill 3 offer higher interface shear strength, allowing the geogrid to efficiently mobilize its tensile capacity without excessive elongation. The failure of fill 1 to meet the 1% strain limit is attributed to its weak shear strength, low stiffness, poor geogrid–soil interaction, and increased creep strain resulting from sustained loads on a compressible fill. These findings indicate that fill 1 is unsuitable for reinforced fill structures where strict geogrid strain limits are required.

### 4.4. Optimization of Geogrid Length in the Selected Model Among Different Solutions

**Figure 15:**
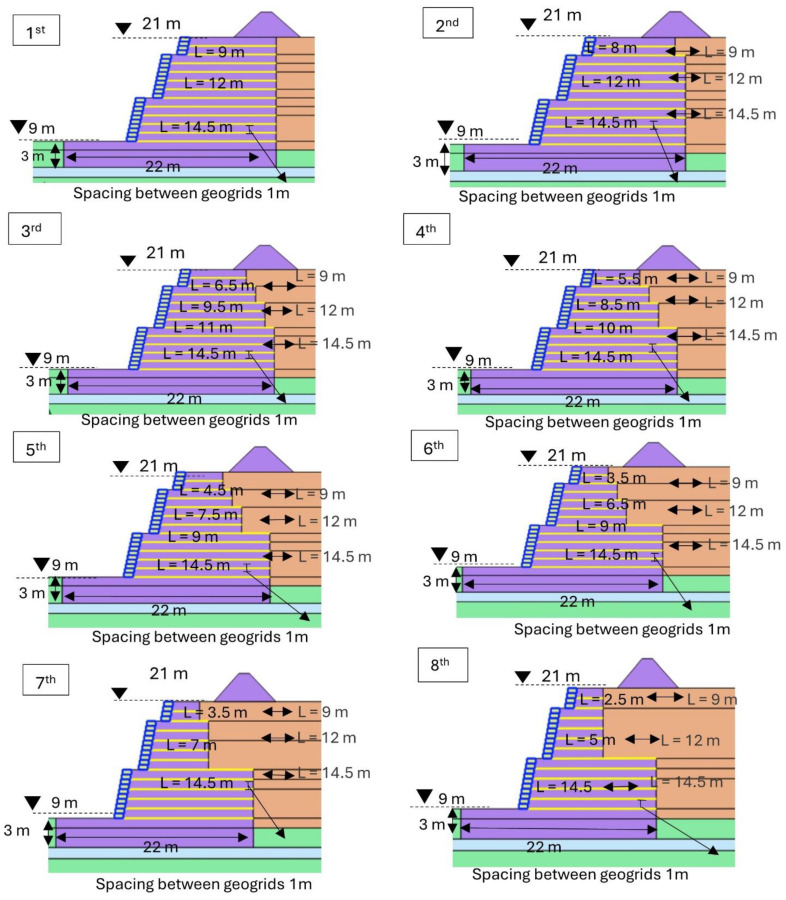
Optimization of geogrid length.

Upon finalizing a viable solution, we further refine it by optimizing the geogrid length, as shown in Figure 15. This optimization aims to achieve a cost reduction while maintaining adherence to specified limits for the safety factor and permissible horizontal displacement and vertical settlement.

**Figure 16:**
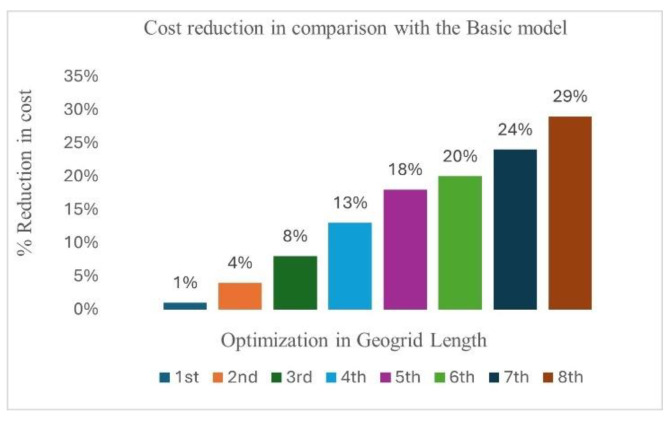
Impact of optimization on cost.

Figure 16 shows the impact of optimization in length of the geogrid on the cost. Without compromising the stability of the structure, we achieved a maximum 29% reduction in the overall cost of the geogrid at the eighth optimization stage. The overall reduction in cost due to optimizing the geogrid length could be realized across various stages, including production, transportation, and installation, thereby making the project more economical. The optimization has very little impact on the horizontal displacement. As shown in Table 11, the horizontal displacement at the toe (Ux) in centimeters is used for various optimization stages. The basic design shows a displacement of 23 cm, and as optimizations proceed from the first through the eighth, there is an observed increase in displacement up to 25 cm, which still lies within our allowable limit and is acceptable. This section may be divided by subheadings. It should provide a concise and precise description of the experimental results, their interpretation, as well as the experimental conclusions that can be drawn.

## 5. Conclusions

Finite element analysis (FEA) was conducted to evaluate the feasibility of three alternative fill materials: weak onsite fill (fill 1), lime-stabilized onsite fill (fill 2), and recycled (C&D) waste (fill 3) for the RFS. The aim was to assess the stability and horizontal displacement of an RFS as well as the long-term creep deformation of the geogrid using a viscoelastic (time-dependent) model in Plaxis to check that the prescribed strain limit of the geogrid conformed to the serviceability limit state.

- The results show that fill 1, with its low shear strength, stiffness, and poor fill–geogrid interaction, leads to an excessive lateral displacement of 85 cm, failing to meet the required stability criteria and making it unsuitable for RFS applications. Additionally, geogrid strain analysis reveals that fill 1 exceeds the prescribed serviceability strain limit (>1%) due to inadequate soil–geogrid interaction and high compressibility;
- With fill 2, the structure remained stable, achieving a Eurocode-based safety factor of 1.12, thereby satisfying stability requirements. The enhanced shear strength and stiffness of fill 2 effectively limited horizontal displacement to 23 cm, while the prescribed serviceability strain limit was within the threshold, thus confirming adequate performance;
- The results for fill 3 demonstrate that the structure remained stable, attaining a Eurocode-based safety factor of 1.19, thereby meeting stability requirements. The higher shear strength and stiffness of fill 3 contributed to a significantly reduced horizontal displacement of 20 cm. Additionally, the geogrid strain remained within the prescribed serviceability threshold, which ensures the effective mobilization of tensile resistance without excessive elongation;
- Finally, an optimization analysis was conducted on the geogrid length using fill 2 to assess its impact on horizontal displacement and cost efficiency. The initial design configuration recorded a displacement of 23 cm. Sequential optimization analysis revealed minimal variation in displacement while demonstrating a clear trend of cost reduction. The cost savings progressively increased, with reductions of 4%, 8%, 13%, 18%, 20%, 24%, and 29% from the first to the seventh stage, respectively. These findings highlight the effectiveness of iterative optimization in reducing costs while maintaining structural performance, reinforcing the importance of systematic design refinement.

These findings demonstrate that both lime-stabilized onsite fill and recycled C&D aggregates provide a viable alternative to conventional backfill materials, ensuring the long-term stability and structural integrity of geosynthetic-reinforced systems.

Future research should include validation of the FEM results with in situ monitoring results to confirm the accuracy. Additionally, future studies should investigate the effects of aging, chemical interactions, and weathering on alternative fills to assess their long-term viability in real-world applications. Real-world testing and additional studies are needed to ensure the results align with actual field conditions and to address any discrepancies that may arise. Moreover, incorporating region-specific cost assessments and economic feasibility studies will enhance the applicability of the cost analysis across different contexts, ensuring its broader relevance and impact.

## Figures and Tables

**Table 1:** Properties of soil for Plaxis-2D (adapted from [40]).

	ϒ_dry_ kN/m^3^	ϒ_wet_ kN/m^3^	Model	C’ref kN/m^2^	Φ’ [°]
Fill 1	17	19	HS	5	25
Fill 2	18	19	HS	20	40
Fill 3	18	19	HS	23	36.8
Clayey sand	18	19	HS	14.7	32
Sand 01	17	19	HS	02	30
Clayey sand	18	19	HS	12	30
Sand 02	19	20	HS	02	32
Clay 02	18	18	HS	08	20
Sand 03	19	20	HS	02	32
Clay 03	18	18	HS	08	22

**Table 2:** Properties of geogrid for Plaxis-2D (adapted from [13]).

Property	Units	Geogrid
Axial stiffness	kN/m^2^	3000
Axial force	kN/m^2^	300
Material type	-	Elastoplastic

**Table 3:** Properties of gabion for Plaxis-2D (adapted from [13]).

Property	Units	Gabion
Unit weight	kN/m^3^	18
Angle of internal friction	Degree	40
Cohesion	kN/m^2^	27
Poisson’s ratio	-	0.3
Elastic modulus	MPa	40
Material model	-	Mohr–Coulomb

**Table 4:** Properties of wire mesh for Plaxis-2D (adapted from [13]).

Properties	Symbol	Units	Value
Axial stiffness	EA	kN/m	62,832
Flexural rigidity	EI	kNm^2^/m	0.251
Weight	W	kN/m/m	0.023
Poisson’s ratio	V	-	0.3
Maximum bending moment	Mp	kN/m/m	0.23
Maximum axial force	Np	kN/m	135
Cohesion	C	kN/m^2^	27

**Table 5:** tan δ/tan φ friction coefficients for wire mesh and geogrid (adapted from [13]).

Soil	tan δ/tan φ (Wire Mesh)	tan δ/tan φ (Geogrid)
Clay	0.3	0.4
Silt	0.4	0.7
Sand	0.65	0.9
Gravel	0.9	0.9

**Table 6:** Material properties of soil for the full-scale wall model for Plaxis.

Property	Symbol	Value
Density	Ρ	1680 kg/m^3^
Angle of internal friction	Ø	44°
Dilation angle	Ψ	11°
Cohesion	C	1 kPa
Poisson’s ratio	v	0.3
Youngs modulus	E	48 MPa

**Table 7:** Material properties of concrete facing for the full-scale wall model for Plaxis.

Property	Symbol	Value
Young’s modulus	E	20 Mpa
Poisson’s ratio	v	0.2
Density	ρ	2500 kg/m^3^

**Table 8:** Material properties of geogrid for the full-scale wall model for Plaxis.

Property	Symbol	Value
Axial stiffness	EA	119 Kn/m
Youngs modulus	E	37.8 Mpa
Poisson’s ratio	v	0.5

**Table 9:** Serviceability limits for post-construction strain.

Structures	Strain
Bridge abutments and retaining walls with permanent structural loading 0.5	0.5%
Retaining walls with no applied structural loading, i.e., transient live loadings only	1%

**Table 10:** Prescribed post-construction strain limit assessment.

	Nmax @ End of Construction (kN/m)	EA Short (kN/m)	Retardation Time (days)	Strain (Short Term) (%)	Nmax @ End of Design Life (kN/m)	EA Long (kN/m)	Strain (Long Term) (%)	Prescribed Post- Construction Strain Limit (%)
	77.42	3160	100	2.45	77.31	1992	3.88	1.43
Fill 1	76.90	3160	200	2.43	76.02	1992	3.82	1.39
	80.55	3160	365	2.55	77.54	1992	3.89	1.34
	16.18	3160	100	0.51	16.31	1992	0.82	0.31
Fill 2	16.38	3160	200	0.52	16.56	1992	0.83	0.31
	16.63	3160	365	0.53	16.09	1992	0.81	0.28
	16.54	3160	100	0.52	16.62	1992	0.83	0.31
Fill 3	16.57	3160	200	0.52	16.52	1992	0.83	0.31
	16.87	3160	365	0.53	16.38	1992	0.82	0.29

**Table 11:** Impact of optimization on horizontal displacement.

	Horizontal Displacement at The Toe UX (cm)
Basic design (First)	23
Geogrid length optimization (second)	24
Geogrid length optimization (third)	24.5
Geogrid length optimization (fourth)	25
Geogrid length optimization (fifth)	25
Geogrid length optimization (sixth)	25
Geogrid length optimization (seventh)	25
Geogrid length optimization (eighth)	25

## Data Availability

The raw data supporting the conclusions of this article will be made available by the authors on request.
